# Scaling up functional traits for ecosystem services with remote sensing: concepts and methods

**DOI:** 10.1002/ece3.2201

**Published:** 2016-06-02

**Authors:** Oscar J. Abelleira Martínez, Alexander K. Fremier, Sven Günter, Zayra Ramos Bendaña, Lee Vierling, Sara M. Galbraith, Nilsa A. Bosque‐Pérez, Jenny C. Ordoñez

**Affiliations:** ^1^ Centro Agronómico Tropical de Investigación y EnseñanzaTurrialbaCosta Rica; ^2^ Department of Fish and Wildlife SciencesUniversity of IdahoMoscowIdaho; ^3^ Departamento de Ciencias AgroambientalesUniversidad de Puerto RicoMayagüezPuerto Rico; ^4^ School of the EnvironmentWashington State UniversityPullmanWashington; ^5^ Thünen Institute of International Forestry and Forest EconomicsHamburgGermany; ^6^ Department of Natural Resources and SocietyUniversity of IdahoMoscowIdaho; ^7^ Department of Plant, Soil, and Entomological SciencesUniversity of IdahoMoscowIdaho; ^8^ World Agroforestry CentreLatin America Regional OfficeTurrialbaCosta Rica

**Keywords:** Ecosystem function, effect traits, functional homogenization, human modification, land cover and climate change, landscape management and policy, LiDAR, regional spatial scale

## Abstract

Ecosystem service‐based management requires an accurate understanding of how human modification influences ecosystem processes and these relationships are most accurate when based on functional traits. Although trait variation is typically sampled at local scales, remote sensing methods can facilitate scaling up trait variation to regional scales needed for ecosystem service management. We review concepts and methods for scaling up plant and animal functional traits from local to regional spatial scales with the goal of assessing impacts of human modification on ecosystem processes and services. We focus our objectives on considerations and approaches for (1) conducting local plot‐level sampling of trait variation and (2) scaling up trait variation to regional spatial scales using remotely sensed data. We show that sampling methods for scaling up traits need to account for the modification of trait variation due to land cover change and species introductions. Sampling intraspecific variation, stratification by land cover type or landscape context, or inference of traits from published sources may be necessary depending on the traits of interest. Passive and active remote sensing are useful for mapping plant phenological, chemical, and structural traits. Combining these methods can significantly improve their capacity for mapping plant trait variation. These methods can also be used to map landscape and vegetation structure in order to infer animal trait variation. Due to high context dependency, relationships between trait variation and remotely sensed data are not directly transferable across regions. We end our review with a brief synthesis of issues to consider and outlook for the development of these approaches. Research that relates typical functional trait metrics, such as the community‐weighted mean, with remote sensing data and that relates variation in traits that cannot be remotely sensed to other proxies is needed. Our review narrows the gap between functional trait and remote sensing methods for ecosystem service management.

## Introduction

**Figure 1:**
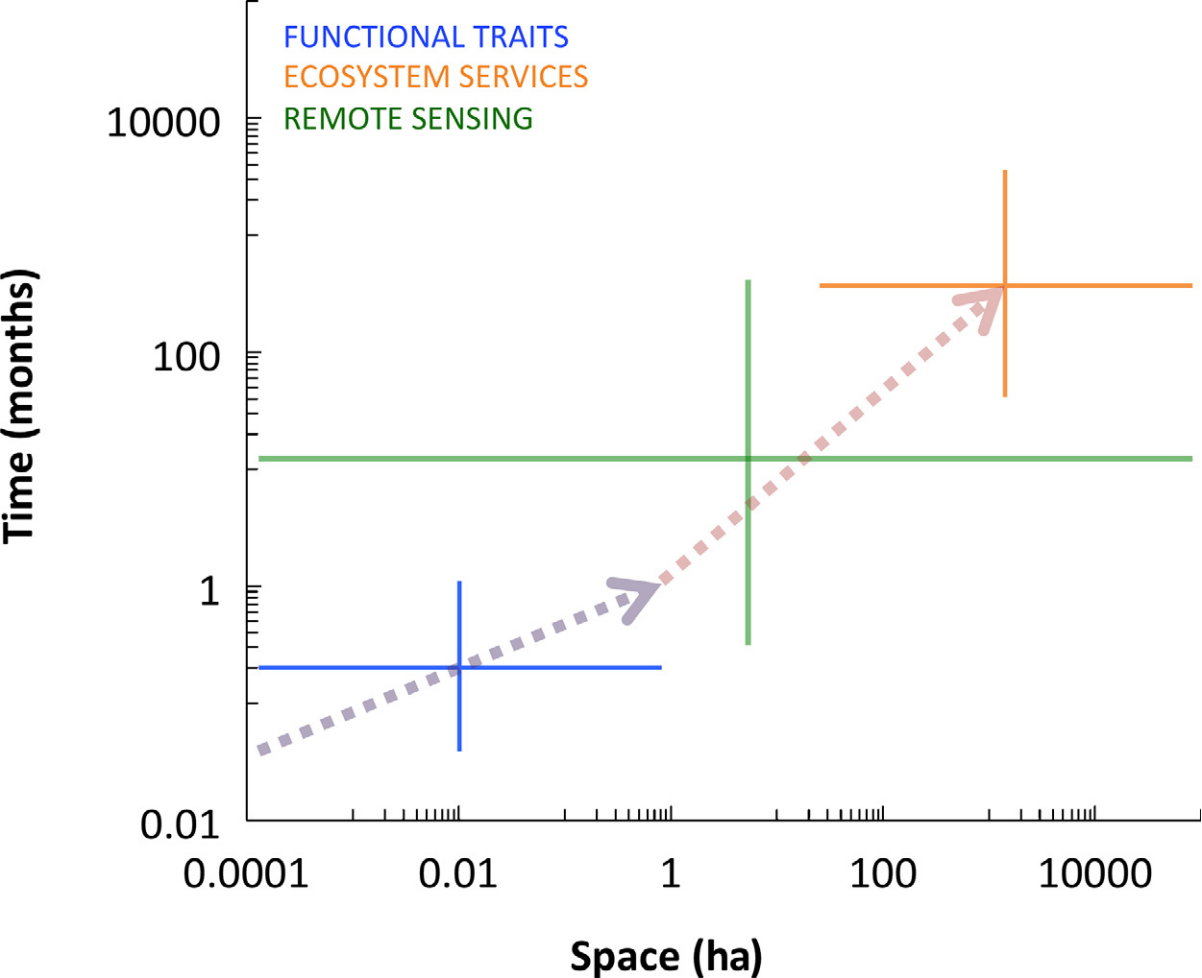
The use of functional traits to inform ecosystem service policy and management requires the scaling‐up of plot‐scale data from local to regional scales. In this review, we outline (1; purple dashed arrow) the sampling considerations for capturing the necessary variation in functional trait composition across space so that these proxies can be used for (2; brown dashed arrow) the fine‐resolution scaling‐up of trait composition from local to regional scales via remote sensing methods.

Evaluation of ecosystem service policy and management requires understanding the consequences of human modification on ecosystem processes and dependent ecosystem services at regional scales (sensu Forman and Godron 1986; Chazdon 2008; Daily et al. 2009). To this end, functional trait approaches have the potential to be more accurate than species‐based approaches due to the continuous nature of functional traits and the direct link between traits and ecosystem processes (McGill et al. 2006; Westoby and Wright 2006). The use of functional traits to inform ecosystem service policy and management requires the scaling‐up of plot‐scale data from local to regional scales (Lavorel et al. 2011). Nevertheless, we currently lack consensus on how to estimate functional trait variation at regional spatial scales relevant to land use planning and policymaking. In this study, our main goal is to review concepts and methods for scaling local plot‐scale functional trait composition to regional scales relevant to ecosystem service policy and management. In particular, we review and synthesize the knowledge necessary for sampling local plot‐level functional trait variation and the available remote sensing methods that can be used to scale up this local trait variation to regional scales (Fig. 1).

Methods for assessing the effects of human modification on ecosystem services typically rely on correlating vegetation type with ecosystem processes (Daily et al. 2009). For example, change from forest to pasture vegetation affects local water fluxes by modifying interception and transpiration (Foley et al. 2003). These local changes can be scaled up to regional scales vis‐à‐vis vegetation type to assess the effects on ecosystem services such as the regulation of peak flood and low flow events (Foley et al. 2007). However, this approach is limited because it does not incorporate the fine‐scale functional trait variation within vegetation types, which can constitute up to 75% of the variation in trait values (Kattge et al. 2011). Traits such as tree biomass, crown diameter, and leaf area affect water fluxes and can vary significantly across and within vegetation types (Meinzer et al. 2005; Park and Cameron 2008; Abelleira 2015). In addition, tree species composition may change but trait composition may not, or vice versa, within a given vegetation type due to trait variation at the individual, species, and community levels (Albert et al. 2010; Messier et al. 2010). Vegetation classifications are also insufficient for determining changes in ecosystem services that depend on the effect traits of highly mobile organisms, whose movement is influenced by habitat heterogeneity and landscape structure (Leyequien et al. 2007; Keitt 2009). For example, traits such as body size affect the foraging range and dispersal capacity of bees, and dependent ecosystem services such as crop pollination (Wray et al. 2014; Martins et al. 2015).

Current research in ecosystem services aims to resolve these issues by focusing on metrics of effect functional traits, such as the community‐weighted mean (CWM) or functional diversity indices, rather than vegetation types (Grime 1998; Lavorel and Garnier 2002; Lavorel et al. 2013). These metrics can be related to the ecosystem processes of interest based on experimental data gathered at local plot scales, and subsequently to dependent ecosystem services according to the values placed on ecosystem properties or fluxes by stakeholders (Díaz et al. 2007; Lavorel et al. 2011; Finegan et al. 2015). Nevertheless, it is unclear how sampling should be conducted to capture the necessary functional trait variation in highly heterogeneous human‐modified regions and whether it is even possible to model the corresponding fine‐resolution trait data at the regional scale with currently available methods (Garnier et al. 2007; Van Bodegom et al. 2012). These concerns limit the adoption of functional trait approaches to quantify ecosystem processes at regional scales relevant for ecosystem service management and policy design (Daily et al. 2009; Fremier et al. 2013; Rollin et al. 2015).

A promising alternative approach is the fine‐resolution regional mapping of functional traits using remote sensing (Fig. 1). Currently available remote sensing methods can provide a direct link between local plot‐scale functional trait variation and regional‐scale ecosystem service management because they are repeatable across time and space, and capable of producing fine‐resolution data across broad areas (Ustin and Gamon 2010; Asner et al. 2011a; Homolová et al. 2013). In particular, remote sensing can facilitate scaling up functional trait variation in highly heterogeneous human‐modified regions where land cover and climate change are disrupting original patterns in trait variation and where ecosystem service assessments are most needed (Daily et al. 2009; Hobbs et al. 2009; Keitt 2009). Although technological advances have improved the available array and capabilities of remote sensing methods, their application to functional trait mapping for assessing ecosystem services at regional scales has not become widespread (Ustin and Gamon 2010; Galbraith et al. 2015).

To better understand the application of functional traits to ecosystem service assessments in human‐modified regions, our objectives are to review (1) the current conceptual understanding of trait‐based approaches for sampling trait variation across spatial scales and (2) existing remote sensing‐based methods that can be used to scale up trait variation from plot to regional scales. We begin our first objective by reviewing the sources of functional trait variation found across ecological levels of organization that span individuals, species, communities, and landscapes. We then synthesize how these sources combine to structure functional trait variation across space in light of human modification, and the implications that the resulting spatial trait variation has for the design of local plot‐scale sampling methods. We structure our second objective around groups of remotely sensible traits that correspond to different sets of available remote sensing methods. In general, phenological and chemical plant traits can be sampled by optical‐based remote sensing while plant structural traits can be sampled by active laser‐based remote sensing. Animal traits may be inferred by combining remote sensing of plant and vegetation structural traits with landscape structure. We illustrate the applicability of some of these methods by citing examples of how variation in plant and animal traits has been sampled and scaled up to regional scales. We end our review by providing a brief synthesis of results, identified knowledge gaps, and outlook for further development of these methods to improve ecosystem service assessments.

## Sampling Trait Variation

### Sources of trait variation

Functional traits can vary across individuals, species, communities, and landscapes. A better understanding of the sources and spatial scales in which most of the effect trait variation is found will allow for better allocation of sampling effort in trait‐based approaches. Although we cannot logistically measure all trait values across all ecological levels, understanding the magnitude of trait variation sources will reduce uncertainty when scaling from local plot‐scale estimates of trait values to broader spatial scales.

#### Individuals and species

Intraspecific variation in functional traits arises from microsite environmental variability and gradients occurring across the geographical range of plant and animal populations (Peat et al. 2005; Bolnick et al. 2011; Violle et al. 2012). Intraspecific trait variation can rival interspecific variation (Hulshof and Swenson 2010; Ruiz‐Jaen and Potvin 2011). The plant trait variation attributable to intra‐ versus interspecific sources can vary by species, trait, or community type (Albert et al. 2010; Kattge et al. 2011; Kazakou et al. 2014). Determining the variation attributable to intra‐ versus interspecific sources in mobile animals is hampered by the challenge of obtaining a fully random sample (De Bello et al. 2011).

#### Communities and landscapes

**Figure 2:**
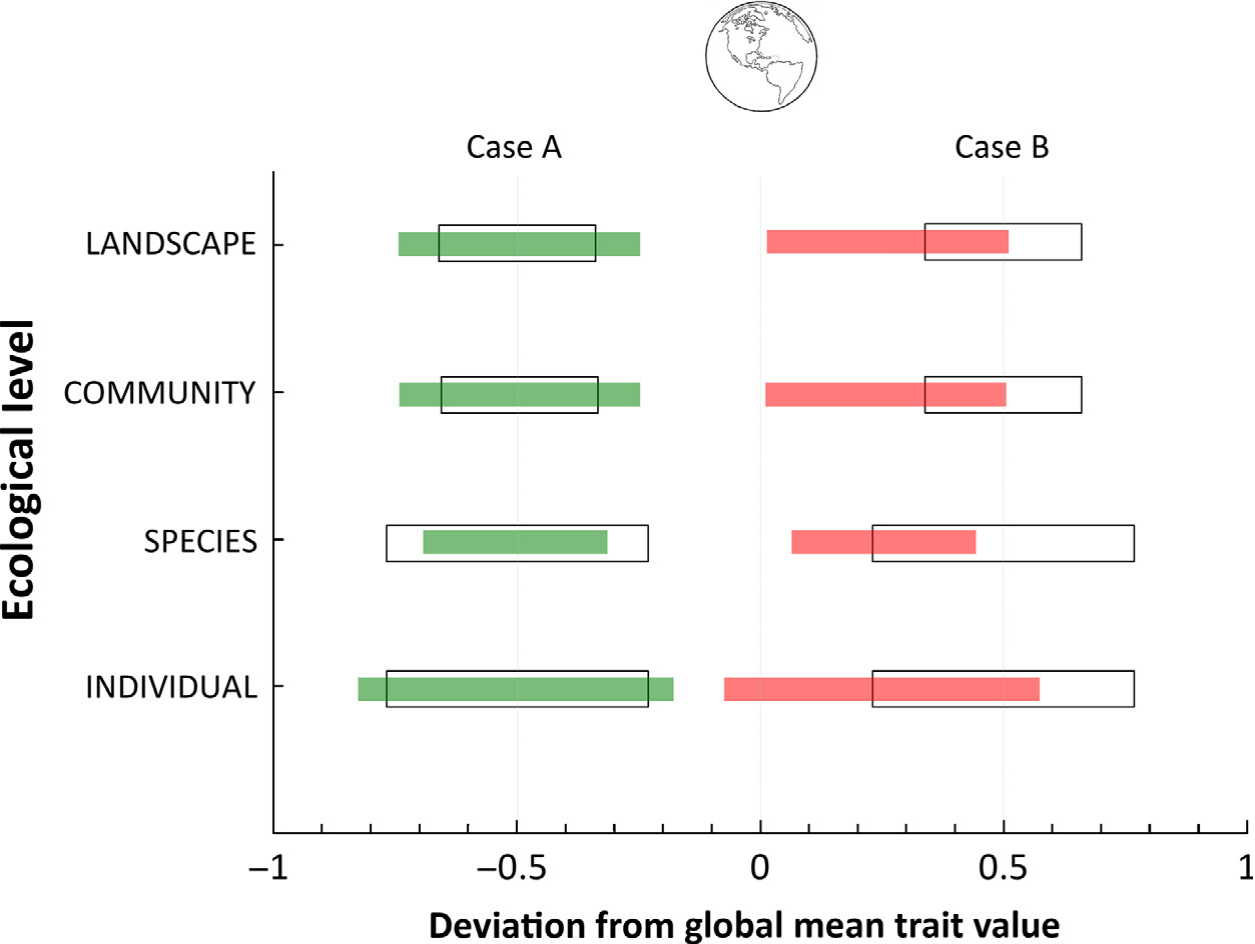
Range of functional trait variation and deviation from a global mean value corresponding to individual, species, community, and landscape ecological levels of a given biome under natural and human‐modified conditions. Black empty bars represent the proportional range of trait variation found across ecological levels under natural undisturbed conditions for two given regions within a biome (hypothetical estimates based on Freschet et al. 2011). Green‐colored bars represent a region (case A) where land cover change and species introductions have resulted in a relative decrease of trait variation found across the species level due to localized extinctions of species coupled with a relative increase in trait variation found across the individual, community, and landscape levels due to the dominance of introduced species, novel and managed community types, and landscape fragmentation, respectively. Red‐colored bars represent another region (case B) where land cover change and species extinctions and introductions have acted to homogenize trait variation by increasing trait values that deviate less from the global mean trait value for the biome at each ecological level. Divergence in trait values between natural and human‐modified regions is higher in case B.

Environmental gradients produced by soil properties, topography, and climate drive functional trait variation across and within natural plant communities (Cornwell and Ackerly 2009; Ordoñez et al. 2009; Swenson et al. 2011; Baraloto et al. 2012). This structuring is less evident at fine spatial scales within communities (≤100 m^2^) due to founder effects and successional processes (Swenson et al. 2007; Yang et al. 2014). Land cover change can disrupt the natural trait variation found within and across plant communities by altering site conditions, successional status, landscape structure, and by species introductions (Fig. 2; Giraõ et al. 2007; Leishman et al. 2007; Lebrija‐Trejos et al. 2010; Lasky et al. 2014). If previous land use intensity and successional stage are accounted for, patterns in trait composition and diversity still emerge across environmental gradients (Mayfield et al. 2005, 2006; Lohbeck et al. 2013). However, there are no consistent patterns in the plant trait composition of successional vegetation across landscapes of varying environmental conditions and land use history (Mayfield et al. 2013).

Overlaying environmental gradients and land use history is the confounding effect of animals on plant trait composition, and vice versa. The functional trait composition of plants can be mediated by the functional diversity of organisms at higher trophic levels and, in turn, have feedback effects on the trait composition of animal communities across the landscape (Giraõ et al. 2007; Cardinale et al. 2012). Pollinators and seed dispersers, which include a wide array of animal groups, affect the trait composition of plant communities in ways that are beginning to be understood (Suding et al. 2008; Lavorel et al. 2013). The trait composition of pollinators and seed dispersers is determined by habitat suitability and landscape structure variables such as patch size and isolation (Tscharntke et al. 2008; Barbaro and van Halder 2009; Bommarco et al. 2010; Jauker et al. 2013). Although the effects of landscape structure on plant trait composition are not as evident as those of environmental gradients and land use history, the loss of pollinator or disperser functional groups due to fragmentation can eventually modify the trait composition of plant communities (Fig. 2; Giraõ et al. 2007; Sutton and Morgan 2009).

### Trait variation across space: impact of human modification

Trait variation is partitioned similarly across intraspecific, interspecific, and community sources, and higher trait variation exists within than across communities due to redundancy across communities (De Bello et al. 2009; Messier et al. 2010; Freschet et al. 2011; Kattge et al. 2011). Less is known about how trait variation is partitioned across spatial scales, yet the steepness of environmental gradients within the spatial extent of interest, rather than the spatial scale itself, appears to drive trait variation (Willis et al. 2010; Freschet et al. 2011; Swenson et al. 2011). Thus, environmental gradients drive functional trait variation across all ecological levels and spatial scales, and natural or anthropogenic disturbances act across levels and scales to counteract trait convergence due to these gradients.

Human modification can alter the trait variation found across ecological levels at the regional scale (Fig. 2). Land cover and climate change can result in the dominance of introduced species possessing traits for which there is no native analog and lead to the emergence of novel community types (Leishman et al. 2007; Hobbs et al. 2009; Abelleira 2011; Drenovsky et al. 2012). Within novel communities, dominant introduced species may increase the magnitude of intra‐ versus interspecific trait variation (Fig. 2; Hillebrand et al. 2008). Managed systems, such as plantations and agriculture, may perpetuate the dominance of introduced species, which can also lead to higher trait variation across community types within a region. The functional traits of novel and managed communities can thus differ from the original communities they replaced and increase the magnitude of trait variation across communities relative to other sources (Fig. 2). Concurrently, functional trait homogenization may occur in regions that have suffered extensive land conversion and high rates of species extinctions and introductions (Olden et al. 2004; Grass et al. 2014). Homogenization may result in trait values closer to the global mean and increase trait divergence between relatively undisturbed and highly human‐modified regions (Fig. 2).

### Implications for trait sampling

Quantifying intraspecific variation may be unnecessary for capturing trait effects on ecosystem processes. However, this variation can be important when one or few species dominate certain community types across environmental gradients (Hillebrand et al. 2008). In such cases, metrics derived from in situ sampling can capture intraspecific trait variation appropriately (Albert et al. 2011). The use of database values for dominant species may miss important intraspecific trait variation effects on ecosystem processes in regions where environmental gradients are steep and few species dominate common community or land cover types (Hillebrand et al. 2008). As species that become dominants are frequently introduced, database values from regions with different environmental conditions may be inaccurate (Drenovsky et al. 2012).

Sampling stratification by community types of varying successional status, novelty, land use history, and management intensity is necessary to capture the effect trait variation in human‐modified regions (Fig. 2; Garnier et al. 2007). Sampling needs to be efficient at capturing cross‐community variation without compromising other trait variation sources. One plot (e.g., ~500 m^2^ for forest tree communities) per site per community type (≥3 sites per community type) at selected points across the prevailing environmental gradients can be enough to capture the necessary effect trait variation into CWM or trait diversity indices (Ackerly and Cornwell 2007; Lavorel et al. 2008; Messier et al. 2010). Dominant species (those contributing >80% of the CWM) should be adequately sampled as outlined by protocols (Cornelissen et al. 2003; Pakeman and Quested 2007). For subordinate species (those contributing <20% of the CWM; Grime 1998) in species‐rich communities, such as old‐growth or mature tropical secondary forests, sampling of one individual per species per plot per site is enough to capture the necessary effect trait variation (Baraloto et al. 2010). In plant communities exhibiting high species dominance and low species richness, database values may be appropriate to estimate effect traits of subordinate species but not of dominant ones (Pakeman and Quested 2007; Lavorel et al. 2008).

At higher trophic levels, landscape structure has greater influence on effect traits by affecting dispersal capacity of highly mobile organisms (Bommarco et al. 2010; Jauker et al. 2013). In the case of mobile animals, CWM and trait diversity indices are typically based on in situ species abundance estimates across community types accounting for variation in landscape structure (Vandewalle et al. 2010). Measuring traits directly among the communities being studied is often not possible due to logistical constraints (e.g., behavioral traits within diverse insect communities). In such cases, traits can be inferred from phylogeny or published keys (Moretti et al. 2009; Vandewalle et al. 2010; Wray et al. 2014). This is more acceptable when trait diversity indices rather than a CWM are used to infer ecosystem services, as the mean trait values in a community may differ from database values depending on variables such as climate and resource availability (Peat et al. 2005; Gagic et al. 2015).

## Scaling up Traits via Remote Sensing

Remote sensing facilitates the scaling‐up of functional trait variation by fine‐resolution mapping of trait‐related data across broad spatial extents. The traits that can be mapped directly with remote sensing are currently limited to plant canopy phenological and chemical traits, and structural traits of plants and vegetation. In general, phenological and chemical traits can be mapped with optical‐based passive remote sensing while structural traits can be mapped with laser‐based active remote sensing. Along with the mapping of land cover and landscape structure, plant phenological, chemical, and structural traits can be related to resource availability for animals in order to infer animal trait variation.

### Canopy phenological and chemical traits

**Figure 3:**
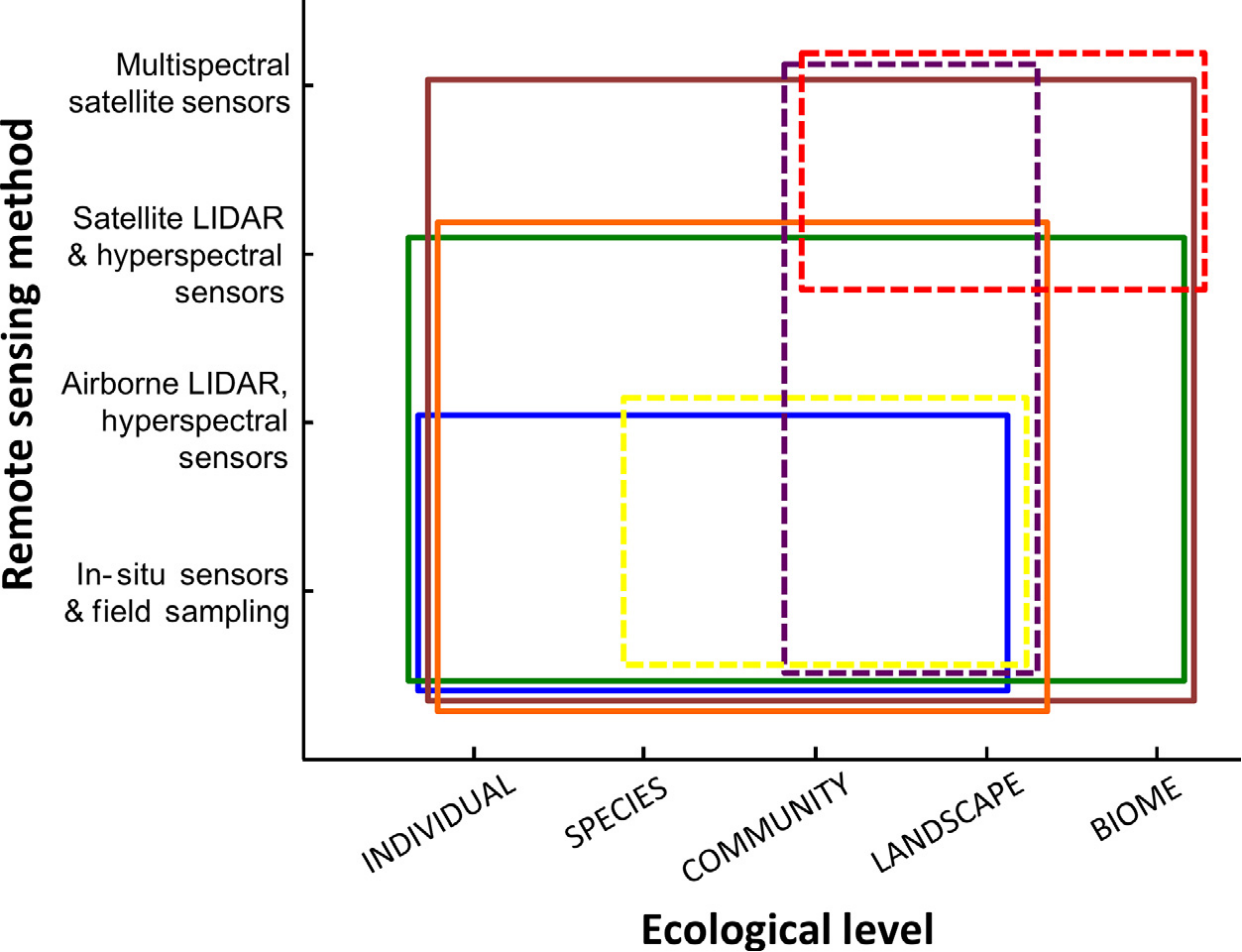
Remote sensing methods for mapping plant and animal functional traits at different ecological levels. The area of the solid boxes covers the ecological levels where remote sensing methods coupled with field sampling and validation allow for the mapping of the following plant functional traits: leaf phenology (brown), leaf chemical content and mass per area (green), plant height (orange), and crown diameter (blue). Dashed boxes cover the ecological levels where remote sensing methods allow for the mapping of the following proxies that relate to animal functional trait diversity: habitat and vegetation structure (e.g., tree density and biomass; yellow), leaf area index (purple), and landscape structure (e.g., patch size, isolation, and perimeter‐to‐area ratio; red).

Plant canopy traits related to phenology and chemistry, such as leafing and flowering periodicity, leaf mass per area, leaf water, carbon and nutrient content, and leaf area index (LAI), can be mapped using satellite‐based passive multi‐ to hyperspectral remote sensing (Fig. 3; Ustin and Gamon 2010; Homolová et al. 2013). Passive sensors such as AVHRR and MODIS have high temporal resolution capable of quantifying the periodicity of leafing and flowering phenology (Fig. 3; Vieira et al. 2003; Kalacksa et al. 2007; White et al. 2009). Landsat can also be used for resolving phenological periodicity due to its spatial and temporal resolution (Kennedy et al. 2012; Melaas et al. 2013). However, most plant canopy phenological and chemical traits cannot be directly retrieved from passive remotely sensed data but inferred by their relationship to canopy spectral properties using empirical or physical models based on statistical relationships or spectral processes, respectively (Gray and Song 2012; Homolová et al. 2013). Empirical and physical models may be used to estimate the spatial variation of similar phenological and chemical traits including leaf mass per area, leaf carbon, cellulose, lignin, nitrogen, phosphorous, photosynthetic pigment, and water content, and LAI and may be used in tandem to facilitate or improve the estimation of other traits (Fig. 3; Colombo et al. 2008; Asner and Martin 2009; Asner et al. 2011b,c).

#### Empirical models

Empirical models use regression analysis to establish statistical relationships between field measurements of traits and passive remote sensing data. A limitation of empirical models has been that most multispectral sensors sample few portions of the electromagnetic spectrum at bandwidths too wide to capture important features for the discrimination of canopy traits (Ollinger 2011). This limitation is addressed by hyperspectral sensors, which sample a high number of contiguous, narrow spectral bands and can be used to derive phenological and chemical traits such as leaf chlorophyll, nitrogen, phosphorous, and water content (Fig. 3; Townsend et al. 2003; Malenovský et al. 2007). With hyperspectral data, the effects of soil background, illumination, or albedo may be reduced with spectral transformations to enhance absorption features of interest (Schlerf et al. 2010). The most important limitation of empirical models is that the relationships between remotely sensed data and field observations of traits can be time‐, site‐, and species‐specific and lack causality, robustness, and transferability across regions (Homolová et al. 2013). Combining data from multi‐ to hyperspectral sensors that capture information at different spatial resolutions and extents (e.g., IKONOS and Landsat) with concurrent ground‐based measurements can help solve this problem (Fig. 3; Anderson et al. 2004; Williams et al. 2008; Gray and Song 2012). The effects of canopy structure on leaf chemical traits are difficult to correct in empirical models, yet a solution is to combine empirical with physical models (Asner and Martin 2008, 2009).

#### Physical models

Physical models of radiative transfer account for light absorption and scattering processes to simulate leaf to canopy reflected or emitted optical spectral properties based on multi‐ or hyperspectral data (Malenovský et al. 2007; Baret and Buis 2008; Jacquemoud et al. 2009; Ollinger 2011). The coupling of leaf and canopy radiative transfer models allows the spectral and directional variation of canopy reflectance to be described as a function of leaf chemistry and canopy structure. This inverse modeling allows for the retrieval of fine‐resolution plant canopy functional traits from plot scale to broader spatial extents (Colombo et al. 2008; Croft et al. 2013; Homolová et al. 2013). Incorporating soil radiative transfer models imposes a strong spectral constraint on the inversion process, decreasing the number of unknown variables and enhancing spectral consistency (Baret and Buis 2008; Jacquemoud et al. 2009). The main drawback of inverse modeling is high uncertainty because several combinations of canopy traits could lead to similar remotely sensed signals (Koetz et al. 2005). Using field data to constrain the distribution and limits of variables can improve the stability and reliability of solutions (Baret and Buis 2008). As radiative transfer models do not incorporate all sources of variability in leaf spectra, the retrieval by inversion is limited to those traits that are directly involved in the modeled process, such as leaf mass per area, chlorophyll content, and water content (Fig. 3; Asner et al. 2011b,c; Homolová et al. 2013).

### Plant structural traits

Individual plant structural traits such as height and crown diameter can be mapped with active remote sensing methods, particularly with LiDAR (Fig. 3; Popescu et al. 2003; Popescu and Wynne 2004; Falkowski et al. 2006; Koch et al. 2006; Popescu and Zhao 2008), LiDAR typically underestimates plant height due to laser returns missing the highest point of tree crowns although the error remains constant (~0.15 m) and is mostly negligible for tall forest canopies (Asner et al. 2012). The sensing of tree and shrub crown diameter with LiDAR remains limited in closed canopy conditions (>50% cover), yet finer postspacing of LiDAR returns (<1 m) may improve the sensing of this trait (Falkowski et al. 2008). Conducting sampling campaigns during the leaf‐off period could also improve tree crown diameter detection with LiDAR in deciduous forests (Brandtberg et al. 2003). Tree crown diameter has been mapped with some success using multispectral aerial photography albeit with the same problems as LiDAR (Strand et al. 2006; Garrity et al. 2008).

Vegetation structural traits such as LAI, tree density, and biomass can also be mapped with LiDAR (Riaño et al. 2004; Martinuzzi et al. 2009; Zhao and Popescu 2009). LiDAR has been used in conjunction with passive remote sensing to scale aboveground carbon stocks in forests from plot to regional and global scales with high accuracy (Asner et al. 2011a; Baccini et al. 2012). Due to its accuracy in sensing forest structure across heterogeneous terrain, LiDAR can be used to map forest type, successional status, and potentially tree species diversity (Asner and Martin 2009; Castillo et al. 2012; Martinuzzi et al. 2013; Hernández‐Stefanoni et al. 2014). Links between plant structure and function can be derived directly from combining the three‐dimensional location data of returned LiDAR pulses with return intensity, which can open new opportunities for fine‐resolution mapping of leaf chlorophyll and N content, and photosynthetic performance (Eitel et al. 2010, 2011; Magney et al. 2014). Improvements in airborne and terrestrial LiDAR technology have increased their utility in characterizing structural traits of low‐stature vegetation such as shrublands and tundra (Streutker and Glenn 2006; Vierling et al. 2012; Greaves et al. 2015). Other active remote sensing methods, such as satellite‐based LiDAR (Lefsky et al. 2005), high‐density laser scanning (Maltamo et al. 2004), and synthetic aperture radar (Santos et al. 2003), can be used to map traits such as canopy height, yet their development lags behind compared to airborne LiDAR (Fig. 3).

### Animal traits

Mapping plant phenological, chemical, and structural traits along with land cover variables, such as successional status and landscape structure, can be useful for predicting the functional traits of animals because they move across landscapes depending on resource availability, and on traits such as foraging range and dispersal abilities (Fig. 3; Leyequien et al. 2007; Jarnevich et al. 2014; Pettorelli et al. 2014). Some optical satellite sensors such as Landsat, IKONOS, and WorldView‐2, which have been typically used for mapping discrete land cover classes and landscape structure, have high enough spatial resolution to resolve the successional status of vegetation (Kennedy et al. 2012).

Vegetation structure variables derived from airborne LiDAR can be used for mapping animal trait diversity by describing horizontal and vertical (three‐dimensional) habitat structure across landscapes. Some useful LiDAR‐derived structural variables include understory vegetation density, LAI, canopy architecture, snag size and density, and tree biomass and basal area (Fig. 3; Turner et al. 2003; Vierling et al. 2008; Bergen et al. 2009; Galbraith et al. 2015). In situ field data on the abundance of animal functional groups can be combined with structural variables to scale up animal trait diversity based on field‐validated models (Hinsley et al. 2002; Martinuzzi et al. 2009; Müller and Brandl 2009; Newton et al. 2009). Coupled with passive sensors that can map leaf phenology and chemistry, the potential for LiDAR to relate plot‐scale structural properties, such as plant canopy height, crown diameter, and aboveground biomass, can facilitate the spatial scaling‐up of multiple plant and animal traits (Fig. 3; Zhao and Popescu 2009; Asner et al. 2011a; Gray and Song 2012; Müller et al. 2014).

## Conclusion

Our review found and synthesized various issues to consider and corresponding viable approaches for scaling up plant and animal traits from plot to regional scales using remote sensing (Table 1). Deciding which of these approaches is more suitable will depend on the traits needed and selected for the ecosystem service assessment. A key issue is the regional context dependency of the relationships between functional trait variation, degree of human modification, and remotely sensed data. Our review shows that functional trait sampling needs to account for the regional modification of trait variation due to dominant introduced species, managed and novel community types, diverse land use history, and heterogeneous landscape structure. Statistical relationships that link local trait variation to regional environmental gradients can fail to capture these anthropogenic effects on trait variation and be of limited use in human‐modified regions. Methods that rely on environmental gradients to integrate field‐sampled functional trait variation into land cover types have been used in ecosystem service assessments (Lavorel et al. 2011). These methods may be significantly improved by applying developments in remote sensing that allow for fine‐resolution regional mapping of trait variation and that directly account for the effects of human modification. Eventually, dynamic vegetation models may reproduce the spatial variation in functional traits after their response to environmental gradients, land cover and climate change (Suding et al. 2008; Van Bodegom et al. 2012). However, the science is not there yet. In situ sampling of trait variation is still needed and more so in highly human‐modified regions for which ecosystem service assessments are most relevant.

**Table 1:** Summary of issues to consider and approaches for scaling up functional traits that resulted from the objectives of this review

Objective	Issues to consider	Approach
1. Field sampling of functional trait variation	Natural sources of plant trait variation are compounded by human modification that results in dominance of introduced species and heterogeneous landscapes	Quantification of intraspecific variation and sampling stratification by successional status, land use history and management intensity may be required
	Land cover change can affect animal traits by modifying the dispersal capacity of mobile organisms	Account for landscape variables related to animal traits, which may be inferred from phylogeny or published keys if necessary
2. Scaling up trait variation via remote sensing	The relationships between plant trait variation and remotely sensed data depend on regional context and more so due to human modification	Remote sensing and ground‐truthing by in situ sampling of trait variation needs to occur independently for regions with different levels of human modification
	Remotely sensed data cannot be directly related to animal trait variation	Animal trait variation may be inferred from the combination of different types of remotely sensed data on vegetation and landscape structure

Our review focused on the objectives of conducting field sampling of trait variation and scaling up trait variation using remotely sensed data with the ultimate goal of improving ecosystem service assessments. An important knowledge gap implicitly found by our review is the lack of research directed toward linking the functional trait metrics that are typically related to ecosystem processes and services, the CWM and functional diversity indices, with remotely sensed data. We could not find any papers on this topic, and this remains a next step to improve the utility of functional traits for ecosystem service management. In addition, most of our review of methods for scaling up traits applied to traits that can be remotely sensed. Other plant traits that may be useful for ecosystem service assessments, such as wood density or belowground biomass, still would need to be inferred based on their relationship to environmental variables or to other traits via modeling approaches akin to the inference of animal traits as illustrated by our review. Nevertheless, fusion of passive and active remote sensing along with technological developments that increase sensor spectral, spatial, and temporal resolutions can improve the mapping of sensible plant functional traits and animal traits related to landscape and vegetation structure. At present, remote sensing is a powerful tool for capturing variation in functional traits at multiple spatial scales and, to improve their accuracy, ecosystem service assessments should take advantage of traits that can be remotely sensed.

## Data Accessibility

All data are included in the manuscript.

## Conflict of Interest

None declared.
